# Downstream Targets of Lmo4 Are Modulated by Cisplatin in the Inner Ear of Wistar Rats

**DOI:** 10.1371/journal.pone.0115263

**Published:** 2014-12-12

**Authors:** Samson Jamesdaniel

**Affiliations:** Institute of Environmental Health Sciences and Department of Family Medicine and Public Health Sciences, Wayne State University, Detroit, Michigan, United States of America; University of South Florida, United States of America

## Abstract

Lmo4, a transcriptional regulator, appears to be a key player in mediating the cochlear pathology in cisplatin ototoxicity, as it controls cellular responses by modulating the formation of transcriptional complexes. We provided the first evidence of in vivo nitration of Lmo4 in cisplatin ototoxicity. Our data suggested that nitration of Lmo4 and associated decrease in its cochlear expression has the potential to play a pivotal role in cisplatin ototoxicity. However, the Lmo4 interactomes that signal the downstream events in the cochlea are poorly understood. Therefore, custom-made gene arrays were employed to evaluate the modulation of known binding partners or targets of Lmo4, in Wistar rats treated with 16 mg/kg cisplatin. RT-PCR analysis, 3 days post cisplatin treatment, indicated that cisplatin induced up/down regulation of multiple cochlear genes associated with Lmo4 signaling. The cochlear expression of Esr1 was significantly up-regulated by cisplatin treatment, while the expression of Stat3 was down-regulated. Co-treatment with Trolox, an otoprotective antioxidant, attenuated the cisplatin-induced modulation of 5 genes in the cochlea. Consistent with the changes observed at the gene level, immunoblots with anti-Stat3 indicated that cisplatin-induced decrease in cochlear protein levels were attenuated by Trolox co-treatment. These results suggest that cisplatin-induced decreases in the cochlear Lmo4 upon nitration, and associated modulation in the cochlear expression of its binding partners Esr1 and Jak1, probably facilitates the repression of Stat3, a downstream target of Lmo4 implicated in drug mediated apoptosis. Collectively, these findings provide insights on Lmo4 downstream events and indicate a potential role of Jak/Stat transcriptional machinery in relaying the Lmo4 protein signaling in cisplatin-induced ototoxicity.

## Introduction

Hearing loss is a major side effect of one of the most frequently used chemotherapeutic drugs, cisplatin. Although considerable progress has been made in delineating the mechanisms underlying cisplatin-induced ototoxicity [1], [2], [3], [4] the components of apoptotic pathways that facilitate cochlear apoptosis are yet to be fully characterized. Previous studies show that cisplatin induces nitration of cochlear proteins, as a strong correlation between dose-dependent increase in cochlear nitrotyrosine and cisplatin-induced hearing loss was observed in cisplatin-mediated ototoxicity [1]. In addition, nitrated proteins localized to cells known to be targeted by cisplatin, particularly outer hair cells. Protein nitration can mediate cellular apoptosis [5], [6] and can cause vital changes in biological function by modulating phosphorylation cascades and altering protein function. Inhibition of cochlear protein nitration, by co-treatment with antioxidant Trolox, attenuated cisplatin-induced hearing loss. We identified the most abundant nitrated cochlear protein as Lmo4, and reported that nitrated Lmo4 was involved in cisplatin-mediated otopathology [1].

Lmo4 is a transcriptional regulator that mediates inner ear development [7], regulates synaptic plasticity in the hippocampus [8], and has been associated with premature aging [9]. Lmo4 has the potential to mediate cytotoxicity, as it controls pathways regulating cell survival and cell death [10]. As a molecular adaptor for protein-protein interactions, Lmo4 controls cellular responses by repressing or promoting transcription [11], [12], [13], [14], [15]. Our studies showed that cisplatin treatment nitrates Lmo4 and decreases Lmo4 expression in the cochlea. Since Lmo4 is considered as a potential mediator of cellular apoptosis [15], the cisplatin-induced regulation of its cochlear expression suggests that it is a plausible target in cisplatin ototoxicity. However, the signaling mechanism by which Lmo4 regulates cisplatin-induced ototoxicity is poorly understood.

Stat3, a downstream target of Lmo4, is a mediator of cell survival [16]. Lmo4 acts as a scaffold to stabilize glycoprotein-130 complex, which facilitates the phosphorylation and activation of Jak1 and leads to the recruitment and phosphorylation of Stat3 [12]. Activation of Stat3 has been reported to promote cell survival by increasing the transcription and cellular expression of anti-apoptotic proteins such as Bcl2 and IAP family proteins [16]. However, cisplatin-induced nitration and decrease in the expression of Lmo4, as observed in our previous study, could eventually disrupt this Stat3-medated anti-apoptotic machinery to facilitate cochlear apoptosis in cisplatin ototoxicity. Therefore, in this study, we evaluated the cochlear distribution of Lmo4, cisplatin-induced modulation of potential Lmo4 interactomes in the cochlea, and cisplatin-induced changes in the expression of Stat3, to clarify the putative Lmo4 signaling mechanism in cisplatin-mediated ototoxicity.

## Methods

### Animals

Three month old male Wistar rats, weighing 0.3–0.35 kg, were obtained from Charles River Laboratories (Wilmington, MA). The animals were housed at the Laboratory Animal Facility and maintained in a temperature controlled room with a 12-h light/dark cycle and allowed free access to food and water.

### Ethics statement

The animals were handled and treated according to the guidelines established by the NIH and the experimental protocol was approved by the Institutional Animal Care and Use Committee of the State University of New York, Buffalo (#HER01072Y).

### Reagents

All reagents were purchased from Sigma (Sigma-Aldrich Corporation, St. Louis, MO) unless noted otherwise.

### Drug administration

Cisplatin was administered at 16 mg/kg body weight dose by slow intra-peritoneal infusion of 1 mg/ml in sterile saline (0.9%) at the rate of 10 ml/h [1]. Control animals were infused with an equal volume of saline. Trolox (100 mg/kg) [1], was mixed with sterile saline (pH 7.2–7.4) and administered by intra-peritoneal injection 1 h prior to and on the 1^st^ and 2^nd^ day after cisplatin treatment. All animals were hydrated with 15 ml/kg subcutaneous injection of saline every day until they were sacrificed 3 days after cisplatin treatment.

### Protein extraction

The animals were anesthetized with CO_2_, decapitated and the cochlear tissues (lateral wall, sensory epithelium and bony modiolus) were dissected in ice cold phosphate buffered saline. The tissue was homogenized in radio-immunoprecipitation assay buffer supplemented with 5 mM EDTA and phosphatase and protease inhibitors (all from Pierce Chemical Co., Thermo Fisher Scientific, Rockford, IL). The homogenate was extracted on ice for 45 min and then centrifuged at 14,000×*g* for 10 min. Protein concentration of the supernatant was determined using the Bradford assay [17].

### Immunohistochemistry

Cochleae were removed from the temporal bone after cardiac perfusion with PBS followed by 10% buffered formalin. Then, three discrete regions of the cochlea (sensory epithelium, lateral wall, and modiolus) were dissected out in ice cold phosphate buffered saline, fixed in 10% buffered formalin for 1 h, and permeabilized with phosphate buffered saline +1% (v/v) Triton X-100 for 30 min. The tissue was blocked for 1 h and incubated overnight at 4°C in Lmo4 primary antibody (Santa Cruz Biotechnology, Inc., Santa Cruz, CA), followed by corresponding secondary antibodies at room temperature for 1 h. Then, f-actin was labeled with fluorescein-conjugated phalloidin. Stained specimens were mounted on slides with ProLong Gold antifade reagent containing DAPI nuclear stain (Invitrogen - Molecular Probes) and examined using a Laser Scanning Microscope LSM 510 Meta (Carl Zeiss, Jena, Germany). Images were captured and analyzed with Zeiss LSM Image Examiner (version 4,0,0,91) [1].

### Westernblotting

Proteins from cochlear extracts were separated on 4–12% gradient NuPage gels (Invitrogen, Carlsbad, CA), transferred to polyvinylidene difluoride membranes, blocked with 0.1% I-Block (Applied Biosystems, Foster City, CA) and probed with Stat3 antibodies (Santa Cruz Biotechnology, Inc., Santa Cruz, CA) using chemiluminescence detection (Pierce Chemical Co., Rockford, IL). A Fuji model LAS 1000 imaging system (Stamford, CT) was used to visualize bands. Background corrected bands were normalized against bands obtained with actin antibodies [1].

### RNA isolation and purification

Cochlea tissue was homogenized in 250 µl QIAzol Lysis Reagent (Qiagen, Valencia, CA). The homogenate was centrifuged at 12,000×*g* for 15 min at 4°C and RNA present in the upper aqueous phase was collected. RNeasy Micro kit (Qiagen, Valencia, CA) was used for purification of total RNA in the lysate. Following manufacturer's protocol, DNA present in the lysate was digested and purified RNA bound to spin column membrane was washed and eluted. The purity of RNA was determined from A260: A230 and A260: A280 ratios.

### Reverse transcription-PCR analysis

RT-PCR was done using Access RT-PCR kit (Promega) following the manufacturer's protocol. Real-time PCR was performed in Bio-Rad MyiQ cycler (Bio-Rad laboratories, Hercules, CA), programmed to include a 45 min step at 45°C followed by a 2 min step at 94°C for first strand cDNA synthesis. Custom-made gene arrays (Cat. No. CAPR10448, SABiosciences) were used to investigate the cochlear mRNA levels of 10 genes (Brca1, Esr1, Stat3, Jak1, Rbbp8, Ldb1, Hdac2, Neo1, Ptpn11, and Pparg) that are known binding partners or targets of Lmo4. These target genes were selected by reviewing the NCBI database for gene interactions and published studies on Lmo4. PCR amplification was done in 40 cycles of a 30 second denaturing step at 94°C, a 1 min annealing step at 60°C, and a 2 min extension step at 68°C.

### Data analysis

Immunoblots were quantified using NIH Image J software. Fold changes of mRNA levels were calculated from Ct values of individual genes using Actb and Rplp as housekeeping genes. The data was statistically analyzed using the GraphPad Prism 5 software. Significant differences between the control and cisplatin as well as cisplatin and Trolox co-treatment groups were determined by one-tailed t tests. In order to illustrate the differences between the cisplatin and Trolox co-treatment groups, the expression levels of individual target genes were normalized to that of the controls. Results are expressed as mean ± standard error.

## Results

### Localization of Lmo4 in three discrete regions of the cochlea

**Figure 1:**
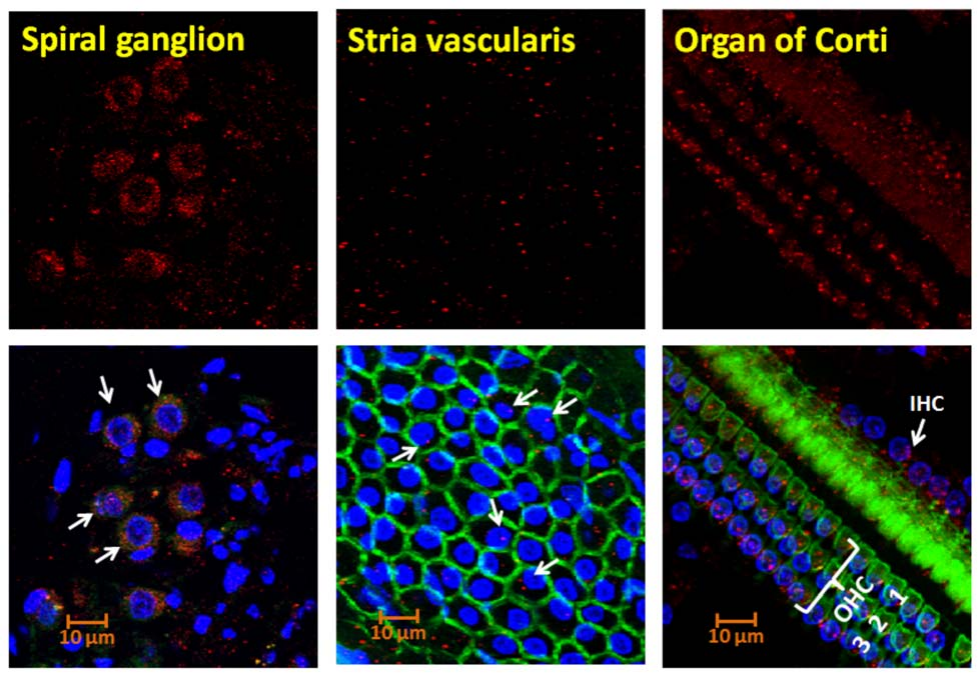
Distribution of Lmo4 in the cochlea. Immunolocalization with anti-Lmo4 detected the expression of Lmo4 proteins in the spiral ganglion, stria vascularis, and organ of Corti of rat cochlea. In the merged panels (bottom row), red indicates immunoreactivity to Lmo4, green indicates actin staining (phalloidin), and blue indicates nuclear staining (DAPI). A section obtained from the basal turn of the organ of Corti is illustrated. The images are representative samples from 2 animals.

The pattern of distribution of Lmo4 in the cochlea indicated that the spiral ganglion and the organ of Corti have a relatively higher expression of Lmo4 compared to the stria vascularis (Fig. 1). Immunostaining in the stria vascularis was sparse and weak. In the organ of Corti, Lmo4 immunoreactivity was detected in the sensitive cellular targets of cisplatin such as the inner as well as outer hair cells and the pillar cells. The stronger staining in the outer hair cells supports the enhanced susceptibility of these cells to cisplatin-induced damage. The specificity of the immunoreaction was indicated by the absence of immunostaining when the specimens were incubated with secondary antibody only (data not shown). Moreover, a single protein band was detected in the western blots when cochlear proteins were reacted with this antibody [1].

### Modulation of Lmo4 target genes by cisplatin

**Figure 2:**
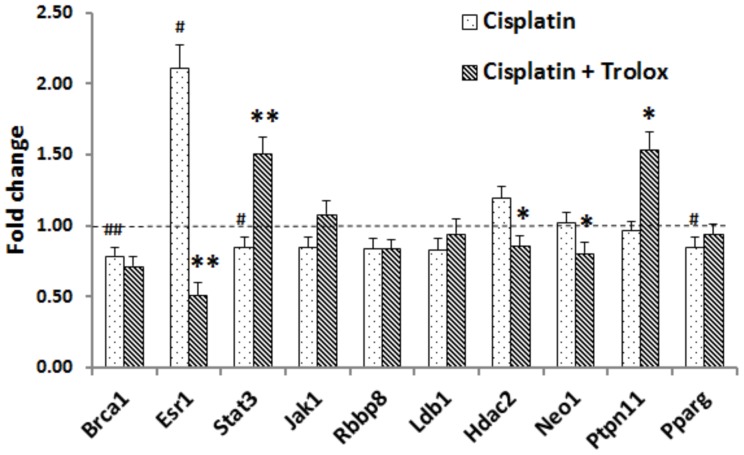
Modulation of Lmo4 binding partners or targets and their attenuation in the cochlea. The graph represents the relative expression levels of 10 genes, after cisplatin or Trolox co-treatment, compared to control animals. Cisplatin treatment altered the cochlear expression levels of 4 genes, which are related to Lmo4 signaling (^#^p<0.05; ^##^p<0.01). The cisplatin-induced modulation of 5 genes were attenuated by Trolox co-treatment (*p<0.05; **p<0.01). Actin and Rplp1 were used as housekeeping genes. Results are expressed as mean ± SE, n = 3.

Cochlear expression of the ten genes that were considered as potential targets of Lmo4, in other models (11,12,13,14,15), was investigated in the cochlea. All ten genes were detected in the control animals by RT-PCR analysis indicating the expression of the Lmo4 target genes in the cochlear tissue. Cisplatin treatment significantly down-regulated the cochlear expression of Brca1, Stat3, and Pparg, and up-regulated the expression of Esr1. Moreover, the cisplatin-induced changes in the cochlear expression of Esr1, Stat3, Hdac2, Neo1, and Ptpn11 were attenuated by co-treatment with Trolox (Fig. 2). Since Stat3 activity has been implicated to play a role in drug-mediated apoptosis (16), it was chosen for further validation.

### Attenuation of cisplatin-induced decrease in cochlear Stat3 levels by Trolox

**Figure 3:**
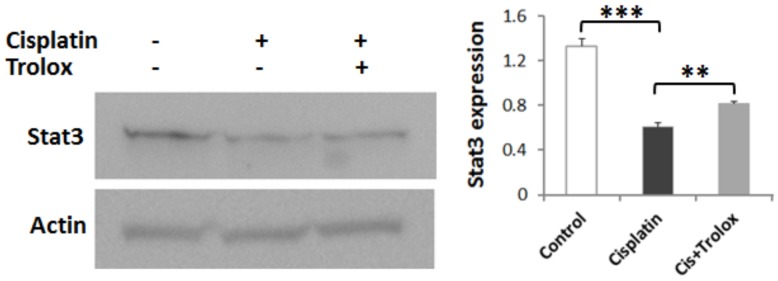
Cisplatin-induced decrease in cochlear protein levels of Stat3. Immunoblots show cisplatin-induced decrease in cochlear Stat3 levels (***p<0.001) and reversal with Trolox co-treatment (**p<0.01). Stat3 expression was normalized with that of actin, and results are expressed as mean ± SE, n = 3.

Cisplatin-induced changes in the protein levels of Stat3 were also in agreement with the PCR results. Treatment with 16 mg/kg cisplatin induced a significant decrease in cochlear protein levels of Stat3, 3 days post-treatment. The cisplatin-induced decreases in cochlear Stat3 were attenuated by co-treatment with 100 mg/kg/day Trolox (Fig. 3), even though the protein levels did not return to that observed in control animals. The specificity of the immunoreaction was indicated by the detection of a single band, which disappeared when the membrane was incubated with secondary antibody only (data not shown).

## Discussion

The efficacy of cisplatin, a crucial life-saving drug used to treat solid tumors, is limited by its major side-effects, namely, ototoxicity, nephrotoxicity, and neurotoxicity. We reported the involvement of Lmo4 and its nitration in cisplatin-mediated otopathology [1]. The present study extends our previous findings by localizing the distribution of Lmo4 in three discrete regions of the cochlea and clarifying the cisplatin-mediated changes in the expression of downstream molecules related to Lmo4 signaling. Particularly, cisplatin induced a down-regulation of Stat3 in the cochlea and the cisplatin-induced changes in the cochlear expression of Stat3 were attenuated by treatment with an otoprotective agent Trolox. These results are in agreement with previous studies that implicate the Jak/Stat pathway in cisplatin-induced ototoxicity [18], [19] and suggests its potential role in relaying the Lmo4 signaling to mediate cochlear apoptosis.

Cisplatin arrests cell division to prevent tumor growth and induces programmed cell death to reduce tumor size [20]. In the inner ear, cisplatin is known to induce oxidative stress in the cochlea, and ototoxicity occurs primarily through apoptosis [3],[21]. Since Lmo4 has been reported to mediate cellular apoptosis and has been identified as the most abundant nitrated cochlear protein after cisplatin treatment, the localization of Lmo4 in discrete cochlear regions is likely to be a critical determinant of the susceptibility of different cochlear cell types to the toxic effects of cisplatin. Previous studies have localized Lmo4 in various stages of the developing cochlea [7]. Here, we report the distribution of Lmo4 in adult rat cochlea by immunolocalizing Lmo4 in the stria vascularis, spiral ganglion, and organ of Corti, which are cochlear targets of cisplatin toxicity. The expression levels in stria vascularis was relatively lower compared to the expression levels in the spiral ganglion, and organ of Corti. Strong signals were detected in the outer hair cells. Since the outer hair cells are considered as a prime target of cisplatin ototoxicity, the distribution of Lmo4 in these cochlear cell types that are sensitive to cisplatin toxicity indicates the critical localization of Lmo4 in the cochlea.

Lmo4 is known to function in conjunction with other proteins in transcriptional complexes and in complexes associated with plasma membrane receptors [11], [12], [13]. In this study, several known target genes of Lmo4 were detected in the cochlea and cisplatin and/or Trolox co-treatment modulated the cochlear expression of a number of them. Although many of these target genes may contribute to the development of the cellular pathology, the cisplatin-induced changes in Lmo4 downstream target Stat3 could facilitate the apoptotic responses as it has been implicated in drug-mediated apoptosis. Moreover, the down-regulation of Jak1 could lead to a decrease in Stat3 activity, because Lmo4 associates with Jak1 and acts as a scaffold to stabilize the glycoprotein-130 complex [12]. This stabilization enables the phosphorylation and activation of Jak1 and Tyk2, which in turn leads to recruitment, phosphorylation, and dimerization of Stat3. However, cisplatin-induced nitration and decrease in Lmo4 levels along with potential down-regulation of Jak1 could destabilize the glycoprotein-130 complex and render it unable to recruit and activate Stat3. Thus, cisplatin-mediated decrease in the expression of cochlear Lmo4 and the tendency to down-regulate its binding partner Jak1 could compromise Stat3 activity and disrupt its anti-apoptotic signaling through this pathway.

Alternatively, cisplatin-induced up-regulation of Esr1 could also compromise Stat3 activity. Esr1 is expressed abundantly in the cochlea [22] and has been reported to facilitate the neuroprotective effects of estrogen in the auditory system [23]. Lmo4 has been reported to bind to Esr1 and repress its transactivation activities [13]. Esr1 physically interacts with Stat3 and inhibits its activity [24]. Because Lmo4 negatively regulates Esr1, a decrease in Lmo4 levels could promote the interaction of Esr1 with Stat3 and facilitate the inhibition of Stat3 activity in the cochlea, which in turn could compromise the transcription of anti-apoptotic genes. Consistent with this hypothesis, cisplatin-induced nitration and decrease in the expression of Lmo4 was accompanied by up-regulation of Esr1 and down-regulation of Stat3, in the cochlea, while these modulations were reversed by treatment with antioxidant Trolox, which attenuated cisplatin-induced nitration of cochlear proteins and prevented associated hearing loss [1].

**Figure 4:**
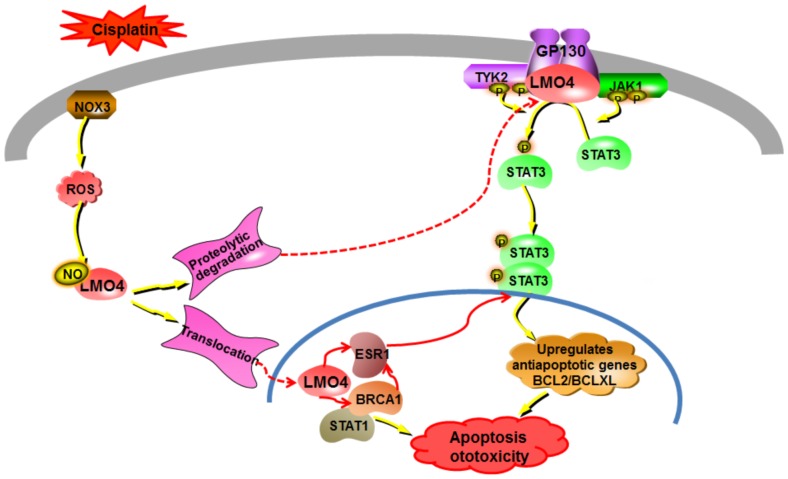
Putative LMO4 signaling in cisplatin-induced ototoxicity. Cisplatin induces cochlear oxidative stress by activating NOX3, which eventually could facilitate the nitration of cochlear LMO4. We reported a cisplatin-induced decrease in the expression of LMO4 in the cochlea (J Biol Chem 2012, 287: 18674–18686). LMO4 has been reported to act as a scaffold by associating with IL-6 receptor glycoprotein 130 and enable the activation of JAK1, TYK2, and STAT3 (J Biol Chem 280: 12747–12757). Since activated STAT3 facilitates the up-regulation of anti-apoptotic genes, the cisplatin-induced decrease in LMO4 levels, probably due to proteolytic degradation of nitrated LMO4, appears to eventually compromise the STAT3-mediated anti-apoptotic machinery, resulting in ototoxicity. Moreover, LMO4 binds with BRCA1 and ESR1 and negatively regulates their activation (Cancer Res 65: 10594–10601). Since BRCA1 directly binds with STAT1, which has been reported to facilitate cisplatin ototoxicity, and ESR1 binds with STAT3 and inhibits its activity, the cisplatin-induced up-regulation of ESR1 and down-regulation of STAT3 in the cochlea suggests that the LMO4 signaling in cisplatin ototoxicity probably involves STAT3-mediated apoptotic pathway to induce cochlear apoptosis.

Inhibition of Stat3 activity has been implicated in drug-mediated apoptosis [25], [26] and activation of Stat3 has been reported to promote cell survival [16]. Therefore, cisplatin-induced decreases in cochlear Stat3 levels and its attenuation after co-treatment with antioxidant Trolox point to the potential involvement of Jak/Stat transcriptional machinery in relaying the Lmo4 signaling to mediate cisplatin-induced apoptotic responses in the cochlea (Fig. 4). Furthermore, cisplatin ototoxicity is attenuated by repressing Stat1 [18], which has an action opposite that of Stat3; this is also consistent with our observations indicating a decrease in cochlear expression of Stat3 after cisplatin treatment. Collectively, these data suggest that Lmo4 might play a pivotal role in cisplatin-mediated cochlear apoptosis. Further delineation of Lmo4 signaling in cisplatin ototoxicity will provide an exciting platform from which to identify new targets for therapeutic intervention, which in turn will enhance the clinical utility of this important anti-cancer drug and improve the quality of life of patients treated with cisplatin.
